# Monitoring environmental impacts of a designated aquaculture area in the Karaburun Peninsula using Google Earth Engine

**DOI:** 10.7717/peerj.20873

**Published:** 2026-02-23

**Authors:** Deniz Devrim Tosun

**Affiliations:** Faculty of Aquatic Sciences, Istanbul University, Istanbul, Turkey

**Keywords:** Aquaculture, Remote sensing, Google Earth Engine, Environment

## Abstract

Satellite-based monitoring of aquaculture impacts remains constrained by the absence of standardized, reproducible methodologies capable of capturing long-term environmental dynamics. This study introduces a novel framework that integrates Difference-in-Differences (DiD) causal inference with multi-decadal Moderate Resolution Imaging Spectroradiometer (MODIS) satellite data and Google Earth Engine (GEE) cloud computing to evaluate aquaculture-related changes in coastal ecosystems. Using 20 years of satellite observations (2002–2022) from the Karaburun Peninsula, İzmir, Türkiye, we compared three representative sites: an aquaculture zone, a coastal area influenced by human settlements, and an offshore reference site with minimal anthropogenic activity. The human-impacted coastal site consistently exhibited the highest concentrations of surface parameters, reflecting dominant background anthropogenic influences. However, DiD analysis revealed no statistically significant differences in chlorophyll-a (Chl-a), particulate organic carbon (POC), or other parameters between the aquaculture and control sites, indicating that potential aquaculture-related effects remained below the detection threshold of the 1 km MODIS resolution. Despite these null results, the study demonstrates the feasibility and limitations of combining causal inference and cloud-based remote sensing for aquaculture monitoring. This methodological integration provides a scalable, cost-effective, and transferable framework for detecting and interpreting environmental change across large spatial and temporal domains. By defining the sensitivity limits of satellite-based detection, this work lays a foundation for future applications that merge high-resolution sensors, *in-situ* validation, and process-based modeling in sustainable aquaculture management.

## Introduction

Aquaculture has emerged as the world’s fastest-growing food production sector, with a phenomenal increase in output over the last 50 years that has been instrumental in meeting the protein demands of a rapidly expanding global population (FAO, 2024). Its ascent has not only diversified our food sources but has also generated substantial economic and social benefits for coastal communities worldwide, providing employment and boosting local economies (Slater et al., 2013; Tosun et al., 2025). However, this rapid and sometimes unregulated expansion has raised significant concerns about its environmental footprint (Skogen et al., 2009; Holmer, 2010). These environmental issues, such as habitat destruction and pollution, have frequently resulted in social conflicts with local communities, who often depend on the same coastal resources (Bush & Marschke, 2016). The central challenge, therefore, lies in balancing the vital role of aquaculture in food security with the critical need to ensure environmental sustainability and long-term ecosystem health for both marine habitats and human communities.

The environmental impacts of aquaculture are widely documented, with known consequences including changes in local water quality, such as nutrient loading, increased turbidity, and altered dissolved oxygen levels (Tovar et al., 2000; Holmer et al., 2008; Ozbay, Blank & Thunjai, 2014; Price et al., 2015). It can also lead to significant alterations in benthic habitats and has the potential to cause eutrophication of coastal waters (Gowen, 1994; Talbot & Hole, 1994; Devlin & Brodie, 2023). However, these impacts are not uniform; they are highly variable and dependent on site-specific factors, including local hydrodynamics, farm management practices, and the scale of operations (Ross et al., 2013; Benetti et al., 2010). This variability makes effective and systematic monitoring particularly challenging. Traditional *in-situ* field surveys, while providing detailed data, are often insufficient for comprehensive monitoring due to their high cost, time consumption, and limited spatial and temporal coverage (Jansen et al., 2016; English et al., 2024). This creates a critical gap in our ability to monitor aquaculture’s broad-scale environmental effects over the long term and across large areas.

In response to these challenges, remote sensing technology has emerged as a cost-effective and efficient alternative for environmental monitoring (Ottinger, Clauss & Kuenzer, 2018; Jiao, 2024). By utilizing instruments mounted on satellites, remote sensing captures crucial data on various environmental parameters, such as sea surface temperature, Chl-a concentrations, and suspended particulate matter (Ramdani, Wirasatriya & Jalil, 2021; Tuzcu Kokal & Musaoğlu, 2021; Sun et al., 2023). This method provides consistent and repeatable measurements over vast areas, enabling the creation of long-term temporal datasets that are unfeasible with traditional field surveys. This study, therefore, utilizes this approach to assess the environmental footprint of a marine aquaculture operation. Our research focuses specifically on a long-term, satellite-derived dataset spanning from 2002 to 2022 to comprehensively evaluate the environmental effects. The study area is located in the Karaburun Peninsula in Izmir, Türkiye, a region designated by the government as an important aquaculture production area, making it an ideal location for a detailed, long-term analysis of aquaculture’s environmental impacts (Tosun et al., 2025).

In recent years, quasi-experimental approaches such as Difference-in-Differences (DiD) have been increasingly applied in environmental and ecological monitoring to isolate intervention-driven effects from background variability. DiD has been used to assess the impacts of marine protected areas, coastal infrastructure development, water-quality regulations, and land-use change, particularly in settings where randomized experiments are infeasible and pre-existing spatial heterogeneity is unavoidable (Shen, Huang & Liu, 2017; Wang et al., 2019). In marine and coastal systems, DiD frameworks are especially valuable for distinguishing localized anthropogenic signals from regionally coherent climatic and oceanographic forcing.

The effectiveness of DiD analyses using satellite-derived environmental data is inherently constrained by the spatial and radiometric resolution of the sensors employed. Previous studies have demonstrated that moderate-resolution sensors such as Moderate Resolution Imaging Spectroradiometer (MODIS) are well suited for detecting regional-scale and long-term surface anomalies, but may fail to resolve subtle or spatially confined impacts associated with localized anthropogenic activities, including aquaculture, particularly when signal magnitudes fall within natural background variability (Le et al., 2013; Tyler et al., 2016). These detection limits underscore the importance of combining robust statistical designs with explicit consideration of sensor sensitivity when interpreting null or weak effects in satellite-based impact assessments.

The main objective of this study is to evaluate the long-term, localized environmental effects of a marine aquaculture operation in the Karaburun Peninsula by analyzing satellite-derived data for key environmental parameters. Our primary hypothesis is that the presence of aquaculture activities has a measurable impact on the surrounding marine environment, which can be observed through changes in sea surface temperature (SST), chlorophyll-a (Chl-a), particulate organic carbon (POC), photosynthetically active radiation (PAR), and normalized fluorescence line height (nFLH).

## Materials and Methods


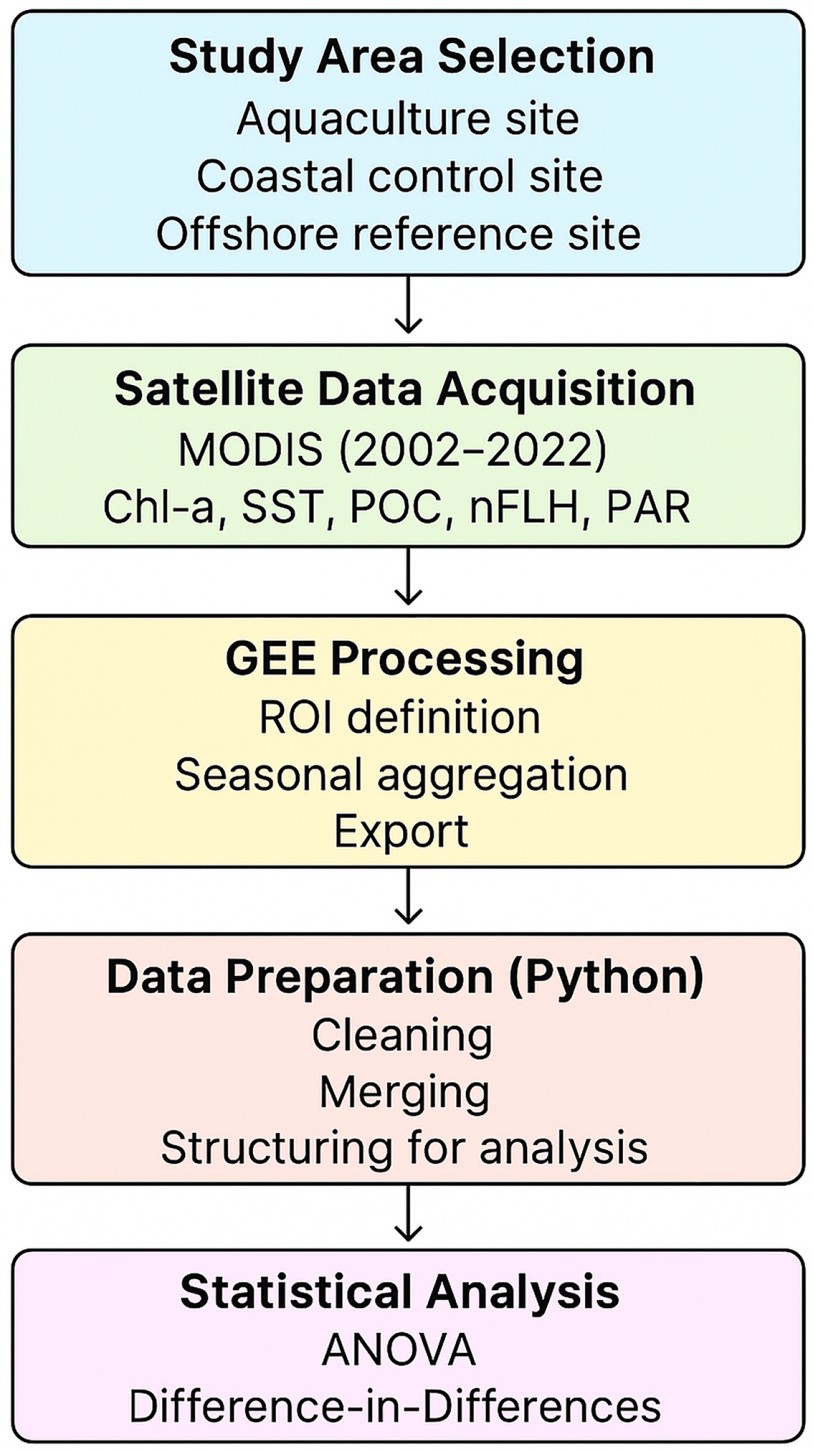


### Study area definition

#### Location

The study was conducted in the **Karaburun peninsula**, situated in **İzmir, Türkiye**. This peninsula forms the extreme western end of Türkiye, as part of the broader Urla-Karaburun-Çeşme Peninsula, and is entirely encompassed within İzmir Province, bordered by the Aegean Sea. Specifically, it is bounded by the Chios Strait to the west, the İzmir Gulf to the northeast and east, and an isthmus stretching between the village of Balıklıova (southeast) and Gerence Bay (southwest). The Karaburun peninsula experiences a warm climate throughout the year. Winter temperatures typically range from 6 °C to 12 °C. In spring, temperatures generally increase to between 16 °C and 22 °C, while summer temperatures consistently exceed 28 °C (Gerçek, 2024). İzmir is a significant contributor to Türkiye’s aquaculture sector; in 2023, Türkiye’s total aquaculture production was 556,287 tons, with İzmir contributing 106,108 tons, making it the second biggest aquaculture producing city (Tepge, 2024). This area is a designated aquaculture region by the Ministry of Agriculture and Forestry.

**Figure 1:**
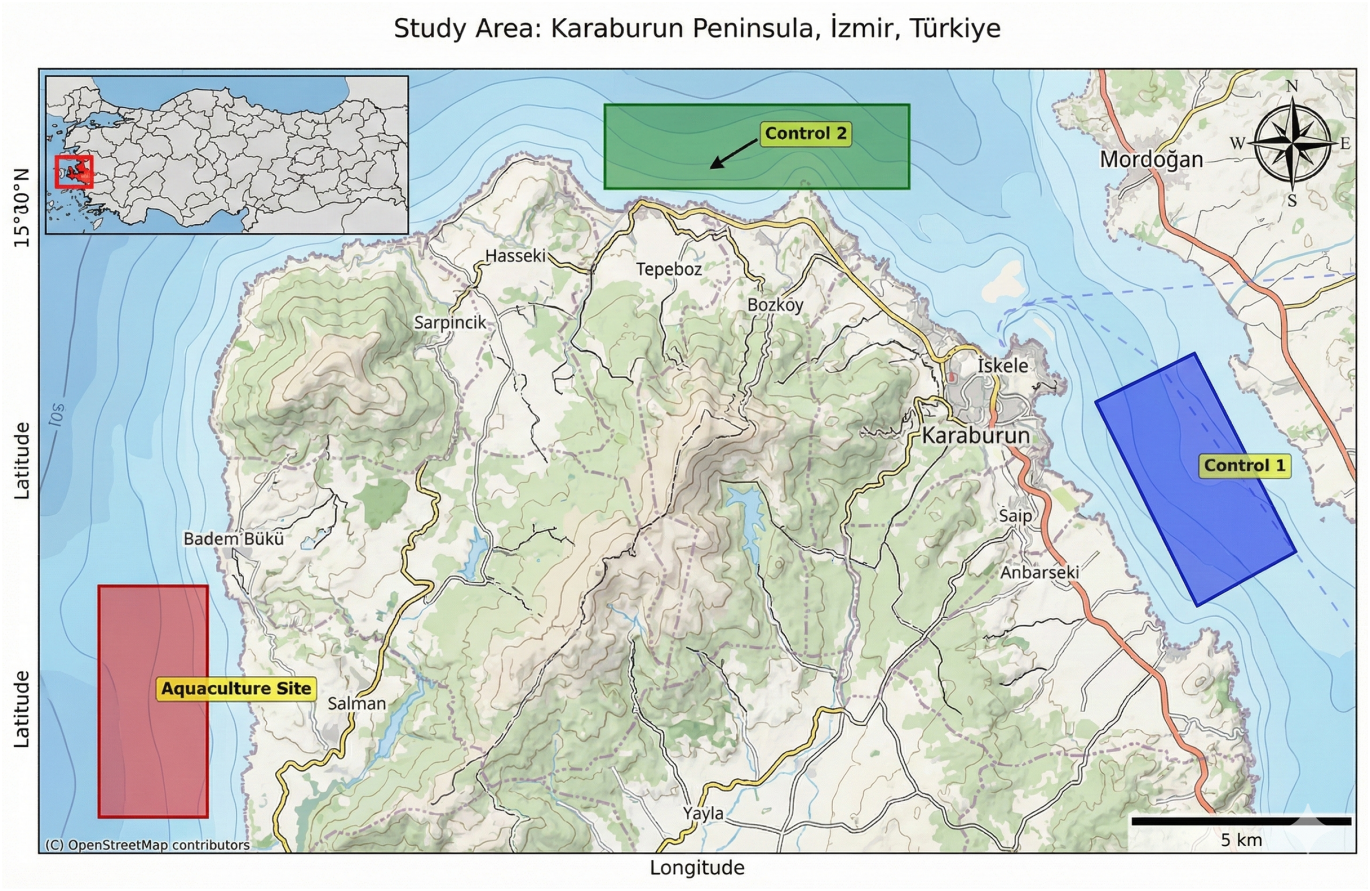
Selected areas for environmental impact comparison.

Three distinct study areas (Fig. 1), each approximately 8 km^2^ in size, were delineated to assess the long-term environmental effects of aquaculture activities using geospatial data from Google Earth Engine (GEE). These areas were selected based on their varying degrees of exposure to aquaculture operations and anthropogenic influence, enabling a comparative analysis over a 20-year period.

#### Aquaculture site

The aquaculture site covers approximately 8 km^2^ (coordinates are embedded in Supplemental Code Files). This spatial extent was defined to represent the full licensed aquaculture production zone rather than individual cage footprints. The area encompasses multiple cage clusters, while control sites were delineated with comparable spatial extent to ensure consistency in spatial sampling.

This scale was deliberately selected to align with the native 1 km spatial resolution of MODIS products, allowing the analysis to capture area-integrated surface signals rather than fine-scale, cage-level effects. Although each site contains a limited number of MODIS pixels, analytical robustness is derived from the long temporal coverage (2002–2022) and seasonal aggregation, which substantially enhance statistical power and support inference on long-term trends and relative changes between sites.

Intensive marine fish farming has been conducted in this area since 2012. It constitutes the sixth designated aquaculture zone established by the Turkish Ministry of Agriculture and Forestry, following a national strategy to relocate nearshore aquaculture facilities to offshore sites with higher carrying capacity and improved water circulation (Akova, 2015). Site selection criteria include water depth, current velocity, and distance from the shoreline, with the objective of enhancing environmental sustainability and reducing spatial conflicts with other coastal users.

Within this designated area, six marine finfish farms are currently operational. Five farms are licensed for an annual production capacity of 2,500 metric tons, while the sixth has a capacity of 2,950 metric tons. The predominant species cultivated are European seabass (*Dicentrarchus labrax*) and gilthead seabream (*Sparus aurata*), with meagre (*Argyrosomus regius*) representing a minor component of production.

Although 2012 was defined as the treatment year corresponding to the onset of offshore aquaculture activities, the transition from nearshore to offshore production occurred progressively rather than instantaneously. The relocation of existing farms and the commissioning of offshore cages followed a gradual ramp-up process, with production capacity increasing over subsequent years. Consequently, any aquaculture-related environmental signal would be expected to emerge gradually rather than as a discrete step change. The Difference-in-Differences framework was therefore applied to assess relative changes before and after the initiation of offshore farming, acknowledging that estimated effects likely represent conservative average impacts over the post-treatment period.

#### Control sites

Two control sites were selected in adjacent areas (Fig. 1). These sites were chosen to be geographically similar to the aquaculture site but free from direct mariculture influence, serving as baseline areas for comparison. The Karaburun peninsula offers regions without settlements or aquaculture facilities, which allowed for the selection of control sites both with and without human settlements to comprehensively evaluate the effects of aquaculture production.

#### Control area 1—human-impacted coastal region (Karaburun Nearshore)

This area is located near the town of Karaburun, a small coastal settlement on the northern coast of the Karaburun Peninsula in western Türkiye. The region lies adjacent to the Gulf of İzmir and is influenced by human activities including residential settlements, tourism infrastructure, and nutrient loads entering from the semi-enclosed gulf, which is known to accumulate anthropogenic pressures from the broader İzmir metropolitan area. Karaburun town, with a population of approximately 12,200 (2022), is situated near the northern tip of the peninsula, facing Foça across the gulf. The region is one of the westernmost inhabited zones of Anatolia and lies north of major tourism centers such as Çeşme. Although free of aquaculture operations, the site’s proximity to both human settlements and hydrodynamically enclosed waters makes it a representative zone for evaluating non-aquaculture anthropogenic impacts. The site covers approximately 8 km^2^ (coordinates are embedded in Supplemental Code Files).

#### Control area 2—offshore reference region (Aegean open waters)

This offshore site is located at the northernmost tip of the Karaburun Peninsula, directly facing the open Aegean Sea. It is considered a relatively pristine reference zone with minimal direct anthropogenic impact and no aquaculture activity. Due to its exposure to strong currents and open-sea conditions, it reflects natural environmental variability and serves as a baseline for ecological comparisons. The site covers approximately 8 km^2^ (coordinates are embedded in Supplemental Code Files).

Colored polygons indicate the spatial extent of each site used for satellite data extraction.

All polygonal regions were delineated and their surface areas calculated using the ee.Geometry.area({‘maxError’: 1}) function within Google Earth Engine. This function accounts for geodesic curvature and provides area measurements in square kilometers, ensuring consistency and spatial precision across all study sites.

### Satellite data acquisition

Data Source: Oceanographic parameters (Table 1) including Chl-a concentration, sea SST, POC, and nFLH were derived from satellite imagery collected by the Moderate Resolution Imaging Spectroradiometer (MODIS) on NASA’s Aqua satellite, specifically utilizing the NASA/OCEANDATA/MODIS-Aqua/L3SMI (Level-3 Standard Mapped Image) dataset (NASA Ocean Biology Processing Group (OBPG), 2022a). PAR data was obtained from a separate MODIS dataset, MODIS/061/MCD18C2 (MODIS Terra+Aqua Daily 3-Hour PAR), where daily mean PAR values were calculated from the original 3-hourly observations (NASA Ocean Biology Processing Group (OBPG), 2022b).

**Table 1:** Summary of satellite parameters.

Parameter	MODIS product	Units	Temporal coverage
Chlorophyll-a (Chl-a)	MODIS-Aqua L3SMI	mg m^−3^	Jul 2002–Feb 2022
Sea surface temperature (SST)	MODIS-Aqua L3SMI	°C	Jul 2002–Feb 2022
Particulate organic carbon (POC)	MODIS-Aqua L3SMI	mg m^−3^	Jul 2002–Feb 2022
Normalized fluorescence line height (nFLH)	MODIS-Aqua L3SMI	mW cm^−2^ µm^−1^ sr^−1^	Jul 2002–Feb 2022
Photosynthetically active radiation (PAR)	MODIS/061/MCD18C2	mol photons m^−2^ day^−1^	Jul 2002–Feb 2022

#### Platform and processing

All datasets were processed on the **Google Earth Engine (GEE)** cloud platform (Ghosh, Kumar & Kumari, 2022; Chen et al., 2023). Custom GEE scripts defined polygonal regions of interest (ROIs) for the aquaculture and control sites. Within each ROI, daily observations were filtered by date and aggregated seasonally (winter, spring, summer, autumn) using ee.Reducer.mean() at the native pixel scale (1 km for MODIS-Aqua products; 5.6 km for PAR). Seasonal mean values and their corresponding year and season were exported as Comma Separated Values (CSV) files for subsequent statistical analysis.

### Data pre-processing

Software: Data pre-processing and statistical analyses were conducted using Python within a Jupyter Lab environment. Standard scientific libraries were used for data manipulation, numerical calculations, visualization, and statistical modeling. Full version information and the complete analysis workflow are provided in the Supplemental Material.

Data Integration: The CSV files exported from Google Earth Engine were organized into structured data tables, harmonized across sites, and assigned to the corresponding aquaculture and control categories. Variable names and data types were standardized to ensure consistency across parameters.

Quality Control: Non-numeric or incomplete entries were handled using conventional data-cleaning procedures to ensure that the datasets used for analysis of variance (ANOVA) and Difference-in-Differences modeling were analytically valid.

Temporal Structuring: A unified time variable was created to support seasonal and long-term analyses. Details of the formatting steps and processing scripts are documented in the Supplemental Material.

### Statistical analysis

Statistical analyses were performed to assess spatial and temporal variations in water quality parameters and to evaluate the potential impact of mariculture activities. A significance level (α) of 0.05 was used for all statistical tests.

#### Descriptive statistics

Summary statistics (mean, standard deviation, quartiles, min, max) were calculated for all water quality parameters, both overall and disaggregated by ‘area_type’ and ‘season’.

Visualizations included two sets of bar plots depicting mean parameter values across area types (with standard deviation error bars): one for parameters with larger value ranges (SST, POC, PAR) and another for parameters with smaller value ranges (Chl-a, nFLH). Additionally, box plots illustrating the seasonal distribution of parameters for each area type were generated, providing a comprehensive visual overview of data characteristics and initial comparisons.

#### Time series trend analysis

Linear regression was employed to identify long-term trends in each water quality parameter for each study area (Aquaculture, Control 1, Control 2) over the 2002–2022 period. The “scipy.stats.linregress” function was used to calculate the slope, *p*-value, and R-squared value for each trend. Results were visualized using heatmaps for both trend slopes and their corresponding *p*-values, facilitating a clear comparison of temporal patterns across sites and parameters.

#### Hypothesis testing (ANOVA & Tukey’s HSD)

To test for significant differences in mean parameter values across the three study areas, a one-way analysis of variance (ANOVA) was conducted using the statsmodels.formula.api.ols and statsmodels.api.stats.anova_lm functions.

Where ANOVA indicated a statistically significant difference (*p* < 0.05), Tukey’s honestly significant difference (HSD) *post-hoc* test (statsmodels.stats.multicomp.pairwise_tukeyhsd) was performed. This allowed for pairwise comparisons between the aquaculture site and each control site, identifying specific significant differences while controlling for the family-wise error rate.

Visualizations included bar plots displaying the mean parameter values for each area type with standard deviation error bars, providing a visual complement to the ANOVA results.

#### Difference-in-Differences (DiD) analysis

To estimate the causal impact of aquaculture activities, a Difference-in-Differences (DiD) regression model was implemented using statsmodels.formula.api.ols. The treatment year was defined as 2012, marking the onset of aquaculture production.

The model was specified as: Y = β_0_ + β_1_·Treated + β_2_·Post + β_3_·(Treated × Post) + ε, where Y represents the water quality parameter, ‘Treated’ is a binary variable (1 for Aquaculture site, 0 for Control sites), ‘Post’ is a binary variable (1 for years ≥ 2012, 0 for years < 2012), and the interaction term (Treated × Post) represents the DiD estimator.

The statistical significance and coefficient of the interaction term were used to infer the unique impact attributable to aquaculture. The analysis relied on the parallel trends assumption, which was initially assessed through visual inspection and subsequently evaluated using formal pre-treatment regression tests.

The parallel trends assumption underlying the Difference-in-Differences framework was formally evaluated using pre-treatment data from 2002 to 2011. Regression models including an interaction term between time and site revealed no statistically significant differential pre-treatment trends between aquaculture and control sites for chlorophyll-a (*p* = 0.469), particulate organic carbon (*p* = 0.410), or photosynthetically active radiation (*p* = 0.992). These results confirm that the DiD analyses are not confounded by divergent pre-treatment trends.

Parallel trends tests were conducted for parameters directly interpreted within a causal Difference-in-Differences framework (Chl-a, POC, and PAR). For SST and nFLH, which primarily reflect regional climatic forcing and optical–physiological variability rather than direct aquaculture-driven processes, formal pre-trend testing was not emphasized, as no causal aquaculture effect was inferred for these variables.

### Software

All data processing, statistical analyses, and visualizations were performed using **Python (version 3.13.4)** within a **Jupyter Lab (version 4.4.4)** environment. Key libraries utilized include Pandas (version 2.3.0) for data manipulation, NumPy (version 2.3.0) for numerical operations, Matplotlib (version 3.10.3) and Seaborn (version 0.13.2) for data visualization, SciPy (version 1.16.0) for linear regression, and Statsmodels (version 0.14.5) for ANOVA and Difference-in-Differences modeling.

### Data availability

The processed time series data for the aquaculture site and both control sites, exported as Comma Separated Values (CSV) files and GEE Script References are available as Supplemental Files.

## Results

This section summarizes the statistical outcomes for five satellite-derived water quality parameters (Chl-a, SST, POC, nFLH, PAR) at the Aquaculture site and two control sites over 2002–2022. To improve clarity, descriptive statistics, long-term trends, baseline comparability, and Difference-in-Differences (DiD) results are summarized using tables and figures.

### Descriptive statistics

**Figure 2:**
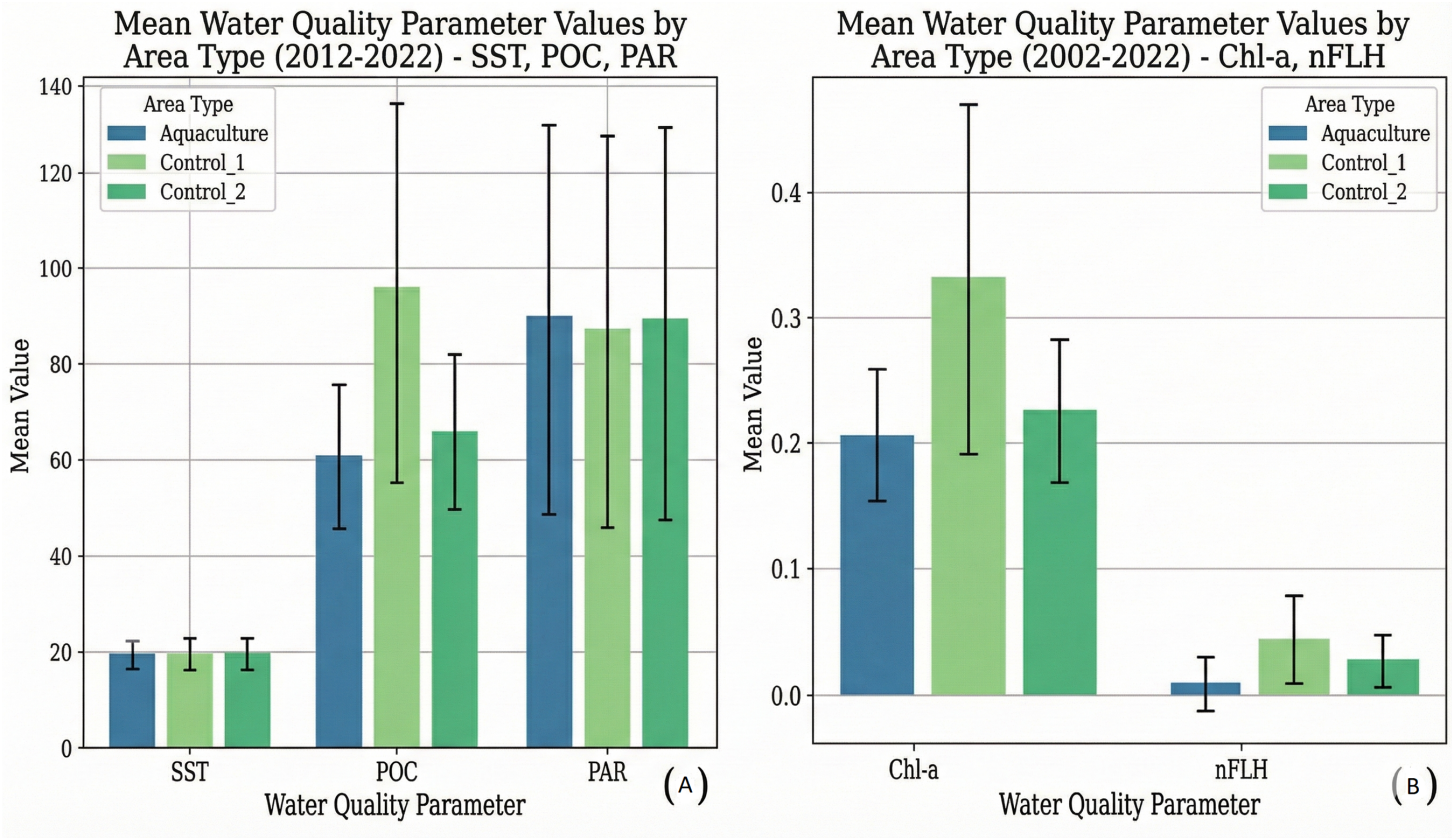
Mean water quality parameter values by area type (2002–2022)—SST, POC, PAR, Chl-a, nFLH. (A) SST, POC, and PAR; (B) Chl-a and nFLH. Bars represent mean values and error bars indicate standard deviation.

**Figure 3:**
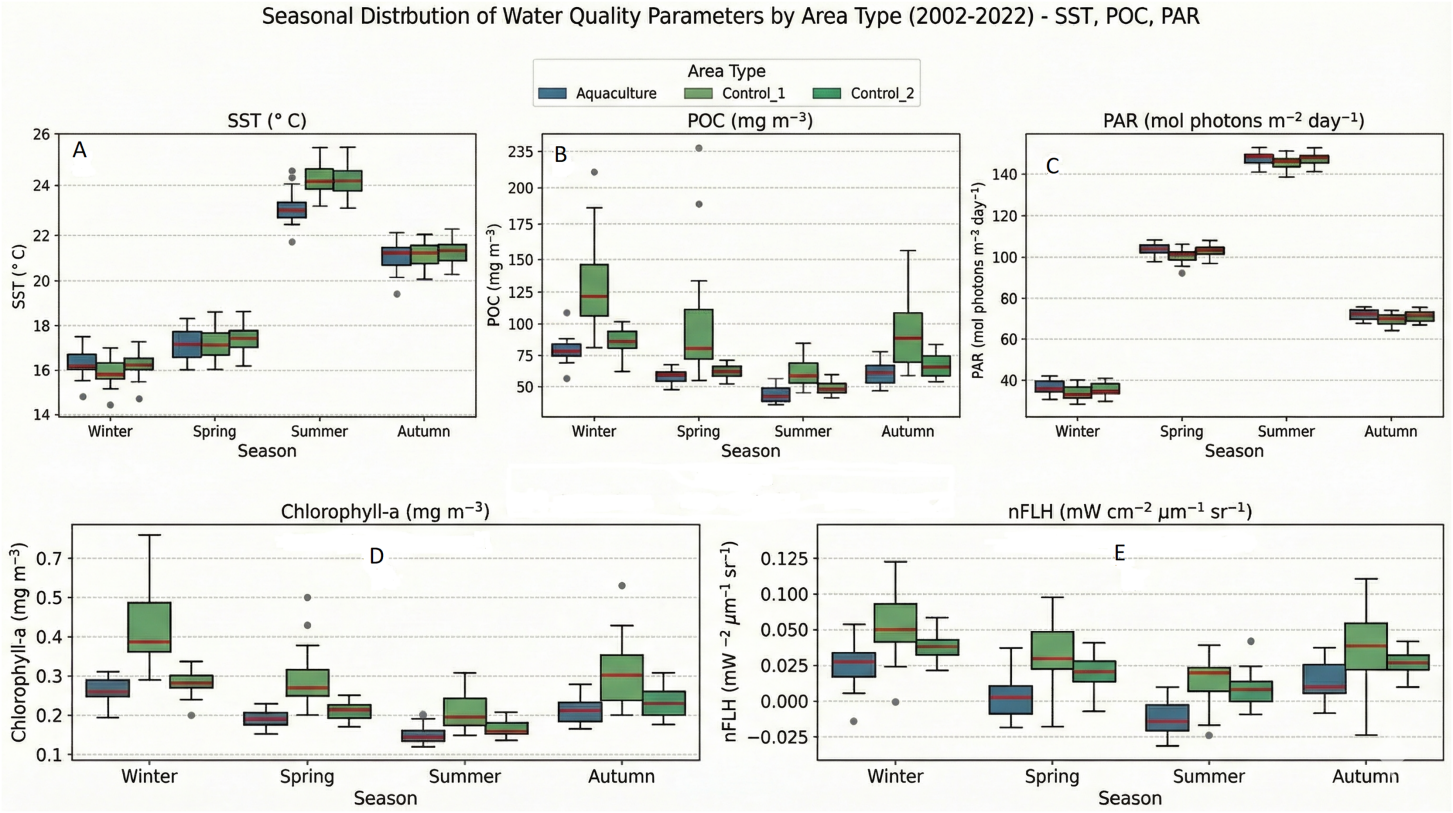
Seasonal distribution of water quality parameters by area type (2002–2022): (A) SST, (B) POC, (C) PAR, (D) Chl-a, (E) nFLH. Box plots show seasonal variability for (A) SST, (B) POC, (C) PAR, (D) Chl-a, and (E) nFLH across the aquaculture site and two control sites.

Overall descriptive statistics for all parameters are provided in Table 2. Mean parameter values for each site are visualized in Fig. 2A (SST, POC, PAR) and Fig. 2B (Chl-a, nFLH). Seasonal distributions are shown in Figs. 3A, 3B.

**Table 2:** Overall descriptive statistics for all parameters (2002–2022).

Parameter	Min	Max	Mean ± SD	Median	Q1	Q3
Chl-a (mg m^−3^)	0.119	0.784	0.254 ± 0.108	0.226	0.181	0.294
SST (°C)	14.467	25.446	19.645 ± 3.149	20.085	16.694	22.240
POC (mg m^−3^)	35.827	227.396	74.141 ± 30.153	66.428	55.253	83.711
nFLH (mW cm^−2^ µm^−1^ sr^−1^)	–0.031	0.144	0.027 ± 0.030	0.024	0.006	0.043
PAR (mol photons m^−2^ day^−1^)	28.347	149.852	88.731 ± 40.717	76.179	42.405	138.072

Across the study area, all parameters exhibited strong seasonal cycles. SST and PAR displayed nearly identical seasonal patterns among all sites, peaking in summer and reaching minimum in winter. POC, Chl-a, and nFLH showed greater spatial variability, with Control 1 consistently exhibiting higher medians and larger dispersion, especially during winter and autumn. Summer values were lowest for POC and Chl-a at all sites, and negative nFLH values occurred frequently in summer, indicating very low phytoplankton fluorescence.

Apart from a slightly lower summer SST at the Aquaculture site, differences among sites were modest for SST and PAR. In contrast, POC, Chl-a, and nFLH showed persistent elevation and variability in Control 1, likely reflecting stronger coastal anthropogenic influence near settlements.

Figure 2A shows that parameters with larger value ranges, such as SST and PAR, exhibited relatively similar mean values across all three sites. Specifically, mean SST was 19.459 ± 2.877 °C for Aquaculture, 19.653 ± 3.347 °C for Control 1, and 19.823 ± 3.233 °C for Control 2. Mean PAR was 89.860 ± 40.683 mol photons m^−2^ day^−1^ for Aquaculture, 87.265 ± 40.912 mol photons m^−2^ day^−1^ for Control 1, and 89.067 ± 41.032 mol photons m^−2^ day^−1^ for Control 2. In contrast, POC displayed its highest mean in Control 1 (95.636 ± 39.676 mg m^−3^), followed by Control 2 (66.066 ± 15.571 mg m^−3^) and the Aquaculture site (60.720 ± 14.759 mg m^−3^).

Figure 2B, focusing on parameters with smaller value ranges, clearly illustrates that mean Chlorophyll-a was notably highest in the Control 1 site (0.331 ± 0.140 mg m^−3^), followed by Control 2 (0.225 ± 0.057 mg m^−3^) and the Aquaculture site (0.206 ± 0.053 mg m^−3^). Similarly, nFLH also showed its highest mean in Control 1 (0.044 ± 0.035 mW cm^−2^ μm^−1^ sr^−1^), with Control 2 (0.027 ± 0.020 mW cm^−2^ μm^−1^ sr^−1^) and the Aquaculture site (0.009 ± 0.021 mW cm^−2^ μm^−1^ sr^−1^) having lower average values.

### Time-series trend analysis

**Figure 4:**
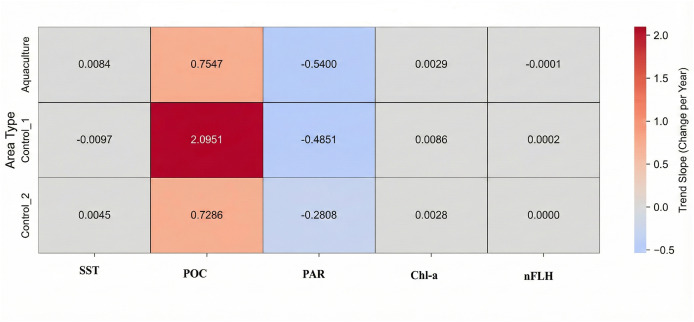
Long-term trend slopes (2002–2022) of SST, POC, PAR, Chl-a, and nFLH across the aquaculture and control sites.

**Figure 5:**
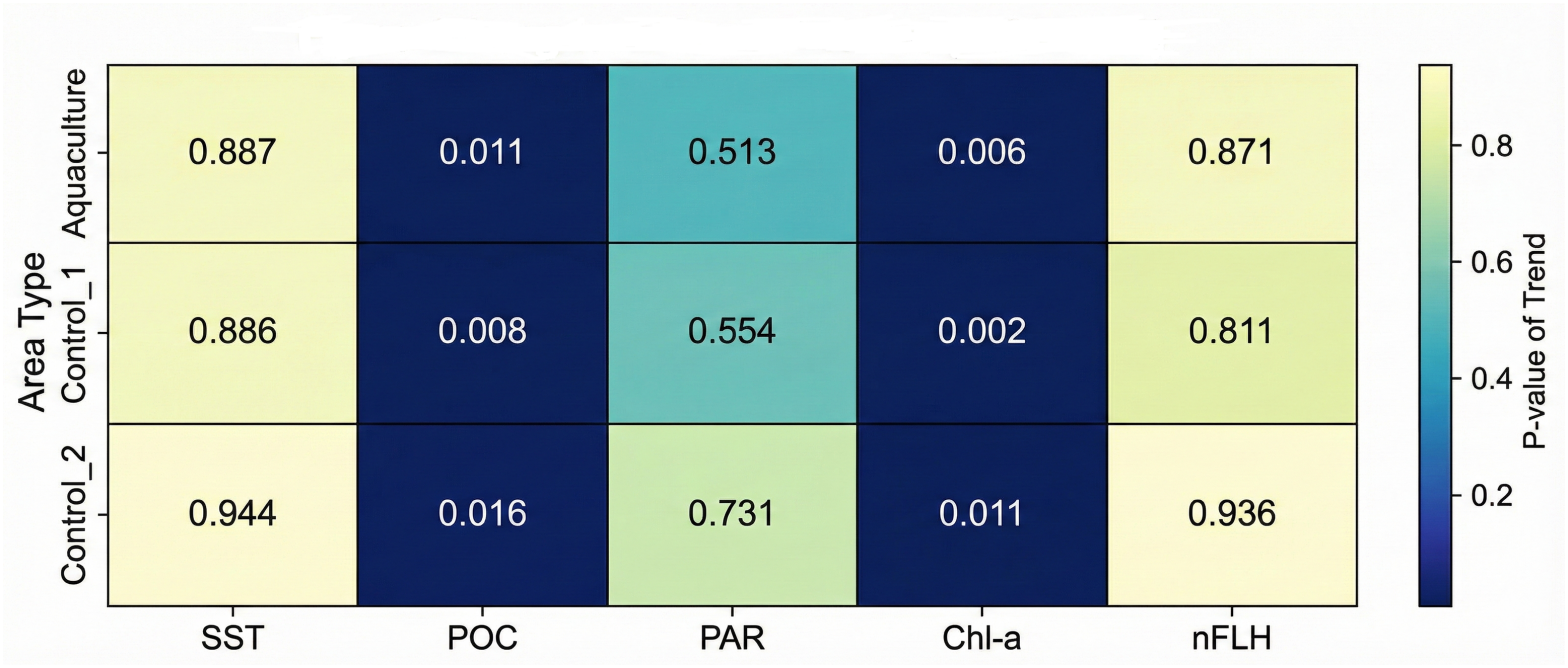
Mean trends for sea surface temperature (SST) by season (2002–2022) at the aquaculture and control sites: (A) Autumn, (B) Summer, (C) Spring, and (D) Winter. The vertical dashed line indicates the onset of offshore aquaculture activities in 2012.

**Figure 6:**
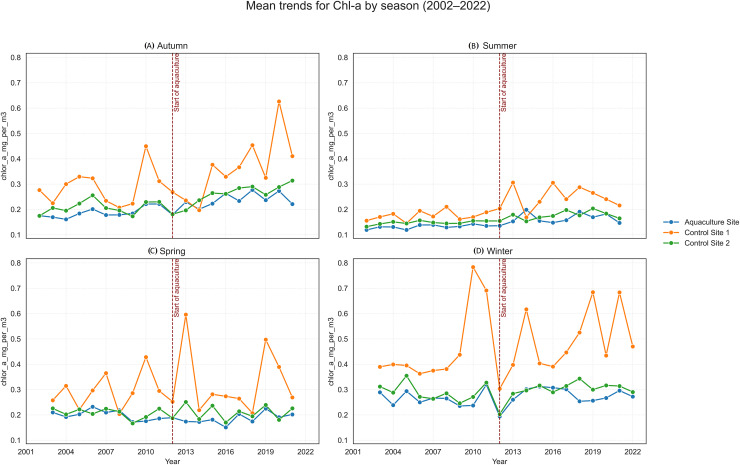
Mean trends for chlorophyll-a (Chl-a) by season (2002–2022) at the aquaculture and control sites: (A) Autumn, (B) summer, (C) spring, and (D) winter. The vertical dashed line indicates the onset of offshore aquaculture activities in 2012.

**Figure 7:**
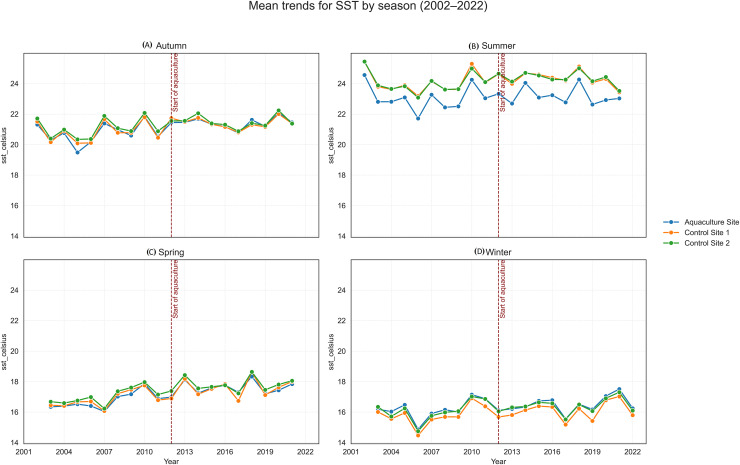
Mean trends for SST by season (2002–2022) at the aquaculture and control sites.

**Figure 8:**
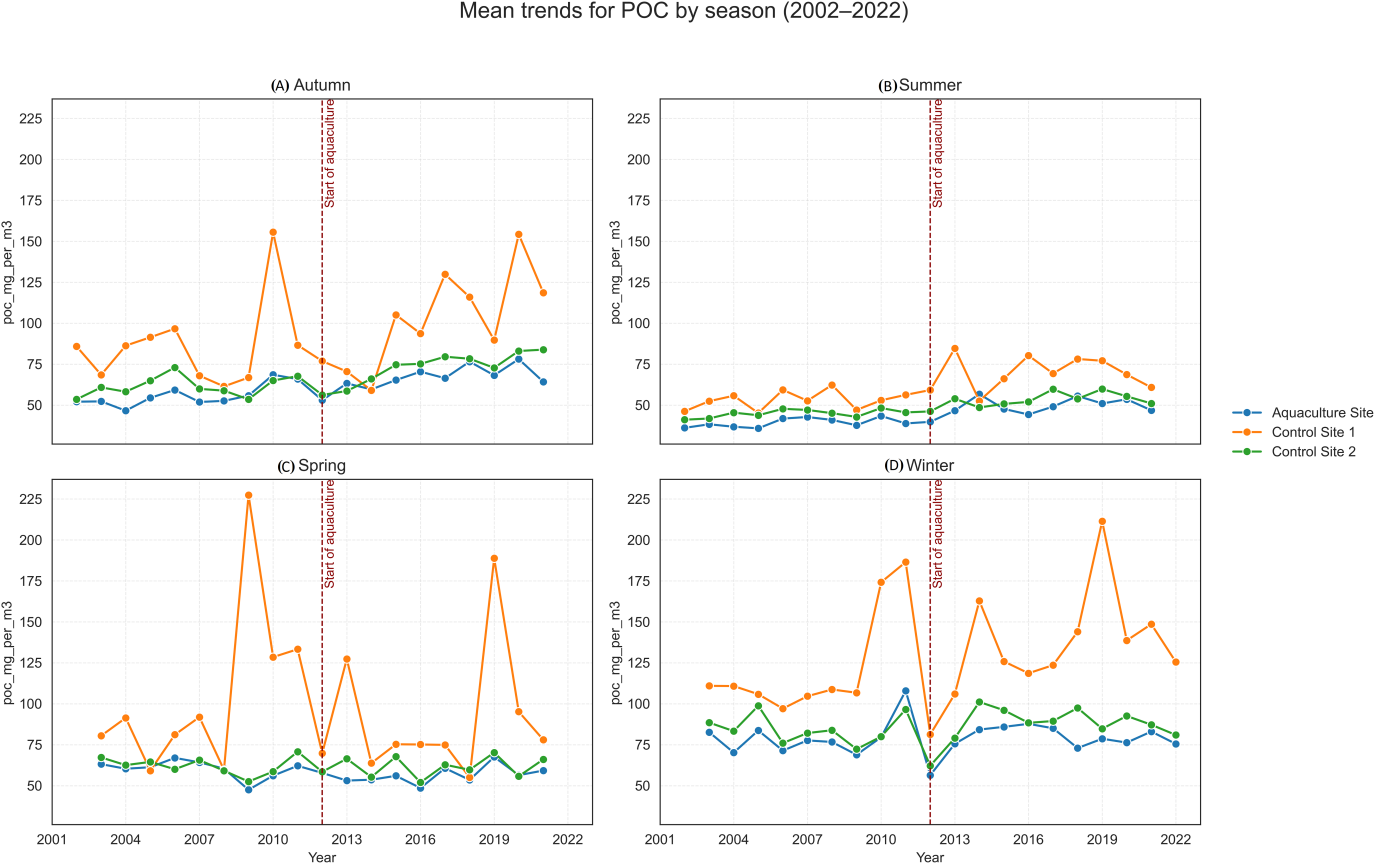
Mean trends for particulate organic carbon (POC) by season (2002–2022) at the aquaculture and control sites: (A) Autumn, (B) Summer, (C) Spring, and (D) Winter. The vertical dashed line indicates the onset of offshore aquaculture activities in 2012.

**Figure 9:**
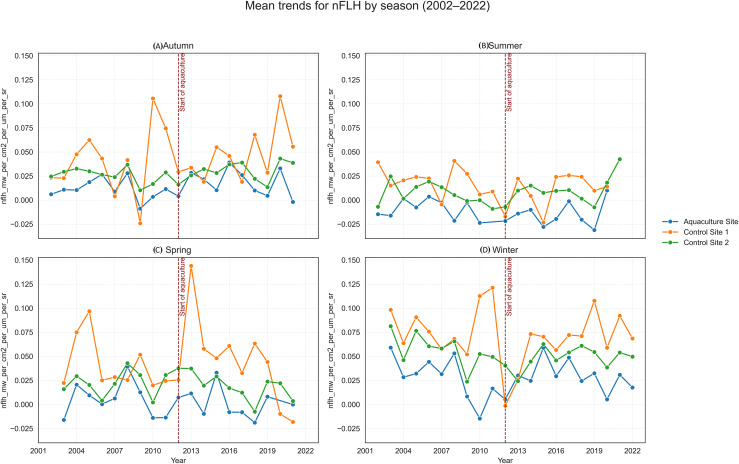
Mean trends for normalized fluorescence line height (nFLH) by season (2002–2022) at the aquaculture and control sites: (A) Autumn, (B) Summer, (C) Spring, and (D) Winter. The vertical dashed line indicates the onset of offshore aquaculture activities in 2.

**Figure 10:**
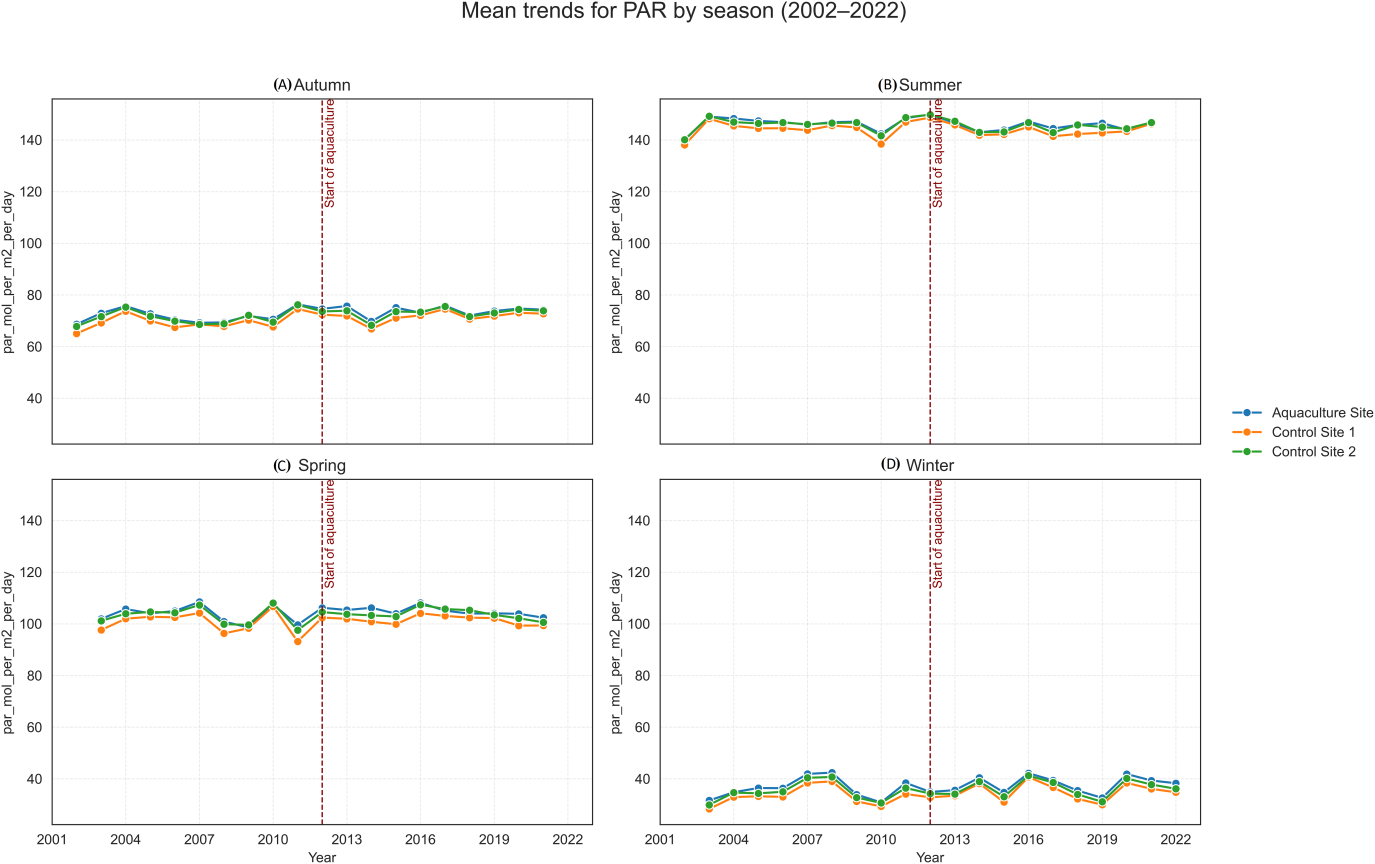
Mean trends for photosynthetically active radiation (PAR) by season (2002–2022) at the aquaculture and control sites: (A) Autumn, (B) Summer, (C) Spring, and (D) Winter. The vertical dashed line denotes the onset of offshore aquaculture activities in 2012.

Long-term linear trends (2002–2022) are summarized in Table 3, with trend slopes and *p*-values visualized in Figs. 4, 5. Seasonal time-series plots (Figs. 6–10) provide the corresponding temporal patterns.

**Table 3:** Long-term trend slopes and *p*-values (2002–2022).

Parameter	Aquaculture (Slope/p)	Control 1 (Slope/p)	Control 2 (Slope/p)
Chl-a	↑ (*p* = 0.031)	↑ (*p* = 0.046)	↑ (ns)
SST	↑ (*p* = 0.002)	ns	ns
POC	ns	↓ (*p* < 0.001)	ns
nFLH	↓ (*p* = 0.047)	↑ (*p* = 0.016)	ns
PAR	ns	ns	ns

SST increased significantly at the Aquaculture site (*p* = 0.002), while no significant SST trends were observed in the control sites. POC showed a strong and significant declining trend at Control 1. Chl-a increased significantly at the Aquaculture site and Control 1, whereas nFLH declined significantly at the Aquaculture site and increased significantly at Control 1. PAR showed no significant long-term trends. Trend lines before 2012 were generally parallel across sites for all parameters, supporting the parallel trends assumption for the DiD analysis.

### Baseline site comparability (Pre-2012)

**Figure 11:**
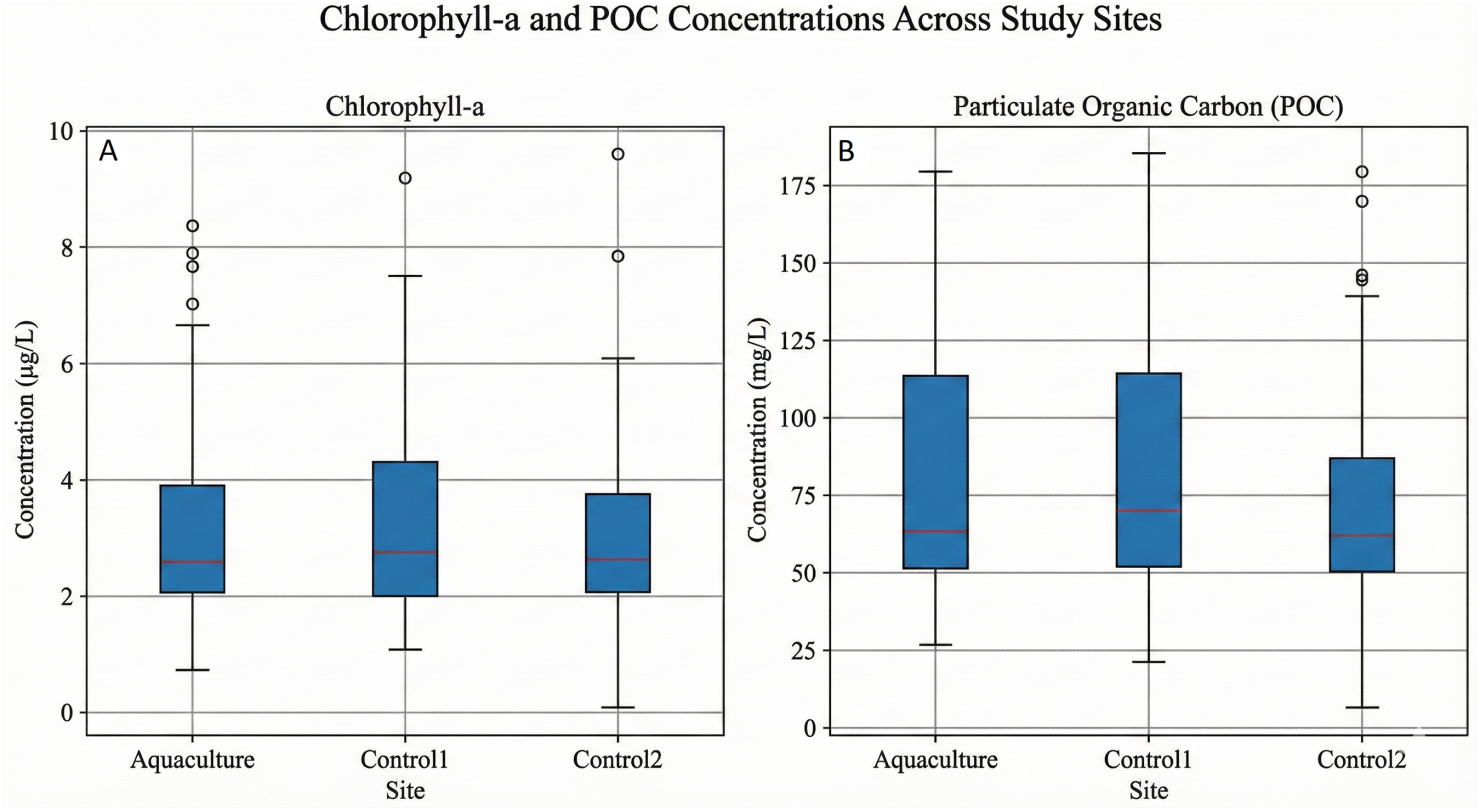
Box plots showing the long-term distribution of (A) chlorophyll-a (Chl-a) and (B) particulate organic carbon (POC) concentrations across the aquaculture site and two control sites, aggregated across all seasons and years (2002–2022). Differences in central tendency and variability among sites are highlighted rather than temporal trends.

To evaluate pre-treatment comparability, an ANOVA followed by Tukey’s HSD was applied to baseline values. Table 4 presents the statistical outcomes, and Fig. 11 shows the corresponding box plots.

**Table 4:** Baseline ANOVA and Tukey’s HSD results (pre-2012).

Parameter	ANOVA F	*p*-value	Significant pairwise differences
Chl-a	42.32	<0.001	Aqua ≠ Control 1
POC	41.20	<0.001	Aqua ≠ Control 1
nFLH	17.14	<0.001	None
PAR	0.08	0.92	None
SST	0.26	0.77	None

Significant differences existed between the Aquaculture and Control 1 sites for Chl-a and POC, while Control 1 and Control 2 did not differ. No significant baseline differences were detected for nFLH, PAR, or SST, indicating comparable starting conditions for these parameters.

### Difference-in-Differences (DiD) analysis

The Difference-in-Differences (DiD) analysis was employed to statistically control for the pre-existing baseline differences in Chl-a and POC identified in the preceding ANOVA. By comparing the changes at the aquaculture site to the natural variations at the two control sites, both before and after the 2012 treatment year, we were able to isolate any unique effect of the farm.

The analysis revealed no statistically significant impact of the aquaculture site on any of the five environmental parameters examined (Chl-a, SST, POC, nFLH, and PAR). Across all four seasons, the *p*-values for the DiD coefficients were consistently above the statistical significance threshold of *p* < 0.05. This indicates that although minor parameter changes were observed at the aquaculture site following 2012, these changes were not statistically distinguishable from natural variability at the control sites.

For example, the DiD coefficient for Chl-a during the autumn season was −0.0135 and was not statistically significant (*p* = 0.7369). Similarly, the relatively large observed change in POC during the spring season (DiD coefficient = 4.6397) also lacked statistical significance (*p* = 0.7896).

As a robustness check addressing baseline heterogeneity concerns, the DiD analysis was repeated using only the offshore reference site (Control 2) for Chl-a, POC, and PAR, yielding results fully consistent with the main analysis (Supplemental).

## Discussion

The environmental impacts of aquaculture have been a subject of extensive research, with numerous studies reporting varied effects on marine and freshwater ecosystems (Primavera, 2006; Cao et al., 2007; Martinez-Porchas & Martinez-Cordova, 2012; Islam & Yasmin, 2017; Bohnes & Laurent, 2021; Jiang et al., 2022). Research has often focused on potential changes in water quality parameters such as dissolved oxygen, nutrient levels, and turbidity, as well as shifts in benthic communities and sediment composition (Tovar et al., 2000; Gamito et al., 2001; Holmer et al., 2008; Ozbay, Blank & Thunjai, 2014; Price et al., 2015). For instance, some studies have documented significant increases in nutrient concentrations and sedimentation near aquaculture sites, attributing these changes to uneaten feed and fish waste (Olsen, Holmer & Olsen, 2008; Kong et al., 2023). However, other research has found minimal to no detectable impact, particularly in sites with high water flow, low stocking densities, or where specific mitigation strategies are in place (Farmaki et al., 2014; Demirak, Balci & TüfekÇi, 2006). This variability highlights the importance of site-specific analysis, taking into account factors like geography, species, and farm management practices. Our study contributes to this body of literature by examining the localized environmental effects of aquaculture operation in the Karaburun Peninsula, İzmir province, Türkiye, using remote sensing geographical data collected over a 20-year period from 2002 to 2022.

The methodology employed in this study, which relies on readily available remote sensing data, presents a highly accessible and cost-effective approach for marine spatial planning. Unlike traditional *in-situ* surveys that are time-consuming and resource-intensive, remote sensing allows for rapid, large-scale assessments over extended periods, like our two-decade timeframe. For decision-makers and regulatory bodies in Türkiye, this method offers a valuable tool for proactive monitoring and for informing the sustainable siting and management of aquaculture operations. It provides a broad, synoptic view that can help identify areas of concern, track long-term trends, and support evidence-based policy decisions without the need for frequent, costly field campaigns.

While our use of a two-decade remote sensing dataset provides a robust long-term perspective on environmental changes, it is important to acknowledge the inherent limitations of this approach. First, remote sensing primarily captures surface-level phenomena, such as changes in Chl-a concentration and SST. Consequently, our analysis is unable to directly measure potential impacts on deeper water column parameters, such as dissolved oxygen levels, or localized changes to the seafloor, including alterations to benthic communities and sediment composition. These sub-surface effects are a common area of concern for aquaculture and would typically require on-site, *in-situ* measurements to assess fully. Therefore, the absence of benthic and sedimentary parameters represents a key limitation of this study and highlights the need for future monitoring programs to integrate satellite observations with seabed-level assessments.

It is important to acknowledge that the treatment and control sites exhibited substantial baseline differences in several environmental parameters, particularly in chlorophyll-a and POC concentrations prior to the onset of aquaculture activities. Although the Difference-in-Differences framework statistically adjusts for such pre-existing heterogeneity, large baseline discrepancies can reduce sensitivity to post-treatment changes and thereby diminish statistical power. It should be noted, however, that the study sites were not expected to be entirely homogeneous before aquaculture began, since the selected areas were already influenced by distinct anthropogenic pressures such as coastal settlements, small-scale fisheries, and tourism activities that predated the establishment of fish farms. This spatial variability reflects realistic environmental conditions along the Karaburun coastline, where true “pristine” control sites are not available.

Given the observed variance in our 20-year dataset, we estimate that any aquaculture-related signal smaller than roughly 5–10% of the natural background variability would fall below the detectable threshold (α = 0.05) with our sample size and temporal coverage. This practical limitation, combined with the coarse 1 km spatial resolution of MODIS, further constrains the ability to identify subtle or spatially confined aquaculture effects. Consequently, the absence of statistically significant results in this study should not be interpreted as evidence of no impact, but rather as an indication that any effects, if present, were below the detection capacity of the data and analytical design used. Future work combining higher-resolution imagery and explicit power analyses will improve detection capability and strengthen causal inference.

Recent advances in high-resolution optical remote sensing, particularly Sentinel-2 imagery, have substantially improved the capacity to detect and monitor fine-scale aquaculture structures and pond-based production systems. Several recent studies have demonstrated the effectiveness of Sentinel-2 and multi-sensor approaches for mapping aquaculture ponds, drainage dynamics, and associated water-quality signals at spatial scales well below 1 km. While such methods are highly suitable for nearshore and land-based aquaculture systems, they are less optimal for long-term regional assessments of offshore production zones, where synoptic coverage and temporal continuity are required. In this context, high-resolution Sentinel-2 analyses should be viewed as complementary to MODIS-based approaches, with future monitoring frameworks benefiting from their integrated use (Hu et al., 2025; Wang et al., 2025).

A *post-hoc* power consideration indicates that, with approximately 80 seasonal observations per site and a significance level of α = 0.05, the analysis is sufficiently powered (≈80%) to detect moderate effect sizes (Cohen’s d ≈ 0.45–0.50). Smaller effects, corresponding to changes below approximately 5–10% of natural background variability, would fall below the detectable threshold. Accordingly, the absence of statistically significant DiD effects should be interpreted as an absence of detectable impacts within the sensitivity limits of the satellite data, rather than evidence of zero effect.

Recent high-resolution remote sensing studies have increasingly emphasized the integration of land–sea processes and fine-scale coastal land-use classification for understanding aquaculture-driven environmental change. In particular, island-specific classification frameworks based on long-term Landsat and Sentinel time series have been developed to resolve the spatiotemporal evolution of coastal land use, including aquaculture ponds and associated infrastructure. For example, Ren et al. (2025) proposed an island-oriented land-use classification system for the Zhoushan Archipelago, highlighting the role of aquaculture ponds as a dominant driver of coastal landscape transformation and environmental pressure.

These advances underscore the growing importance of high-resolution, land–sea integrated approaches for nearshore and island systems. However, such frameworks are primarily optimized for pond-based or land-adjacent aquaculture and are less suited to long-term, offshore monitoring where synoptic coverage and temporal continuity are critical. In this context, moderate-resolution sensors such as MODIS remain valuable for detecting regional-scale surface signals and long-term variability in offshore aquaculture zones, while high-resolution island- and pond-focused approaches should be viewed as complementary tools rather than substitutes.

Second, the observational and correlational nature of remote sensing data means that while we can identify correlations between aquaculture activities and environmental changes, we cannot definitively establish a causal link. The Karaburun Peninsula is a dynamic coastal region, and other potential factors, such as regional climate variability, tourism, and runoff from land-based activities, could also contribute to the observed changes. While our long-term dataset helps to mitigate the influence of short-term weather events, it remains challenging to isolate the specific impact of aquaculture from these other confounding variables. These regional-scale drivers are therefore acknowledged as major confounding factors that may obscure or interact with potential aquaculture-related effects.

Finally, while the temporal and spatial resolution of our remote sensing data is a key strength, cloud cover and other atmospheric conditions may have resulted in gaps in the dataset for certain time points. These gaps, although minimal, could potentially obscure some intermittent changes or short-term environmental fluctuations.

Multiple parameters and seasonal trend tests were conducted; however, these analyses were intended to provide descriptive context rather than formal hypothesis testing. No correction for multiple comparisons was applied, and individual trend significance values should therefore be interpreted cautiously. Importantly, the primary inferential results of the study are based on the Difference-in-Differences framework, which yielded no statistically significant aquaculture effects and is not affected by multiple comparison issues.

Throughout the parameter-specific interpretations below, observed spatial differences and temporal patterns are discussed as descriptive signals rather than statistically confirmed effects. Unless explicitly supported by Difference-in-Differences results, these patterns should be interpreted as sub-detectable variability that remains below the statistical resolution of the satellite-based analysis.

### Key environmental parameters and their significance

Our analysis focused on a set of key environmental parameters derived from remote sensing data, each providing unique insights into the potential effects of aquaculture on the surrounding marine environment. The present study focused on a suite of five key satellite-derived parameters SST, Chl-a, POC, PAR, and nFLH to evaluate the environmental impact of aquaculture. We acknowledge that our dataset did not include direct measurements of water column turbidity or suspended particulate matter (SPM). However, the influence of these factors is implicitly reflected in our findings. The resuspension of sediments and the introduction of waste particles from aquaculture sites are the primary causes of increased turbidity, a condition that is directly correlated with elevated levels of POC. Furthermore, high turbidity can significantly reduce light penetration, which in turn directly impacts both PAR and the photosynthetic activity of phytoplankton, as indicated by our Chl-a data. Therefore, while not a directly measured parameter, the relationships observed within our data underscore the indirect effects of turbidity and highlight its importance as a key variable for future studies to consider.

Chl-a is a key indicator of phytoplankton biomass and is often used to assess nutrient enrichment and potential eutrophication near aquaculture sites (Doering, Chamberlain & Haunert, 2006; Zang et al., 2011). Waste inputs from farms can elevate Chl-a, making it a central parameter for evaluating ecosystem responses (Zhang et al., 2006; Eze et al., 2021).

Our findings align with studies reporting limited phytoplankton responses in well-flushed environments. For example, nutrient enrichment in Monastir Bay (Tunisia) had minimal trophic effects due to strong hydrodynamic mixing (Challouf et al., 2017). Similar open-water conditions in the Karaburun Peninsula likely dilute farm-derived nutrients and help explain the absence of Chl-a increases in our analysis.

By contrast, enclosed or weakly mixed systems often show clear eutrophication signals. In Lake Nasser, Egypt, Chl-a concentrations were consistently higher within farm areas than in adjacent waters (El-Otify, 2015), illustrating how limited circulation promotes nutrient accumulation.

These comparisons highlight the importance of hydrodynamics in shaping aquaculture impacts. In our study, mean Chl-a differences among sites did not reach statistical significance in the DiD framework, indicating that they reflect background variability rather than measurable aquaculture-driven change.

Sea surface temperature is not a direct indicator of aquaculture-derived pollution, but it is a fundamental physical variable shaping marine ecosystem processes (Baldock et al., 2014; O’Carroll et al., 2019; Mengual, Sanchez-Jerez & Ballester-Berman, 2021). Aquaculture structures may induce small, localized thermal effects by altering water movement, although farms are typically more influenced by ambient temperature variability than they are drivers of it (Martinez et al., 2018; Kim et al., 2021). Long-term SST patterns are therefore essential for interpreting ecological responses, as warming trends can affect the physiology of wild and farmed organisms and modify community structure (Parra et al., 2018; Mengual, Sanchez-Jerez & Ballester-Berman, 2021; Verma et al., 2022).

In our study, SST exhibited consistent seasonal cycles across all sites, indicating shared regional forcing. The aquaculture site showed slightly higher mean temperatures than the control areas, which may reflect minor local modifications to circulation around farm structures. However, the overall SST dynamics were dominated by broader climatic patterns, serving primarily as a contextual baseline rather than evidence of aquaculture-driven thermal change.

Particulate organic carbon functions as a direct indicator of suspended organic matter (uneaten feed and fecal waste) originating from aquaculture operations (He et al., 2024). Elevated POC near farms can reflect nutrient loading and waste accumulation, making it a key parameter for assessing localized water-quality impacts. Intensive fish farming is known to release particulate C, N, and P into the surrounding environment through feed loss and defecation, influencing pelagic and benthic systems (Mente et al., 2006; Wang et al., 2012). These inputs can stimulate phytoplankton and macroalgal biomass when hydrodynamic conditions allow for retention (Olsen & Olsen, 2008).

In our study, the aquaculture site showed higher POC levels relative to the control sites, aligning with previous observations of localized waste dispersal from fish farms (Kotta et al., 2023; Elvines et al., 2024). This pattern also corresponds with the slightly reduced PAR values at the aquaculture site, as increased suspended material can diminish light penetration. Importantly, while the POC signal suggests a localized accumulation of organic matter, corresponding Chl-a and nFLH patterns indicate that this did not translate into broader eutrophication.

However, the Difference-in-Differences results showed that these POC differences were not statistically significant. Accordingly, they should be interpreted as descriptive indicators of localized waste influence rather than evidence of a confirmed farm-driven impact.

Photosynthetically Active Radiation represents the portion of sunlight available to support primary production, forming the base of marine ecosystem functioning (Gao et al., 2012). Aquaculture installations and increased turbidity from suspended particulate matter can diminish light penetration, with potential implications for primary producers and overall ecosystem productivity (Gattuso et al., 2006).

In this study, PAR values were consistently lower at the aquaculture site than at both control sites over the 20-year period. When interpreted alongside the elevated POC concentrations, this reduction is coherent with the expected effects of farm-derived organic material increasing turbidity and limiting light availability. This mechanism aligns with the conceptual framework described by Sahoo & Anandhi (2023), who note that suspended particles and nutrient inputs from anthropogenic sources can reduce underwater light transmission.

The persistent PAR depression at the aquaculture site therefore points to a sustained, localized physical effect (likely driven by turbidity) rather than a nutrient-driven shift in phytoplankton biomass. This distinction reinforces the interpretation that the farm’s primary environmental signal in the water column is physical light attenuation, not widespread eutrophication.

nFLH offers a sensitive optical proxy for phytoplankton fluorescence and provides a more robust indicator of physiological status than standard Chl-a retrievals, particularly in optically complex coastal waters (Behrenfeld et al., 2009; Gower & King, 2012; Gower, Young & King 2013). Because it minimizes interference from non-photosynthetic particles, nFLH helps disentangle biological signals from turbidity and other confounding factors (Moloto et al., 2023).

In this study, nFLH patterns closely paralleled those of Chl-a, indicating moderate phytoplankton biomass that was not elevated relative to the control sites. This congruence supports the conclusion that the aquaculture operation did not induce broad-scale eutrophication. Instead, the nFLH record reinforces the interpretation derived from Chl-a and POC: any effects are localized and primarily physical (*e.g*., turbidity-driven), rather than indicative of nutrient-driven phytoplankton proliferation.

Thus, nFLH provides an independent and more diagnostically precise confirmation that phytoplankton dynamics near the farm remain within the bounds of natural background variability rather than reflecting an aquaculture-driven bloom signal.

## Conclusions

Our long-term satellite-based analysis provides valuable insight into the environmental dynamics surrounding aquaculture operations in the Karaburun Peninsula, Türkiye. Chl-a concentrations within the aquaculture site were generally lower than in the nearshore control area and comparable to the offshore reference site. This indicates that nutrient enrichment from aquaculture did not produce statistically significant or widespread eutrophication effects, and that the natural variability in coastal productivity is a dominant driver in this region.

Slightly elevated POC and reduced PAR were observed near the aquaculture site, yet these trends were not statistically significant. They may represent small-scale, localized responses below the detection threshold of the 1 km MODIS sensor. This highlights the spatial resolution limitations of MODIS data in capturing fine-scale aquaculture signatures, particularly in hydrodynamically active open-sea environments.

Overall, our results suggest that aquaculture contributes to minor, localized variations in surface parameters, but its detectable footprint is much smaller than the broader anthropogenic pressures such as urbanization, tourism, and land-based runoff that affect the Karaburun coastal system. While the Difference-in-Differences analysis detected no statistically significant aquaculture effects, descriptive patterns suggest the possibility of minor, localized variability that remains below the detection threshold of the satellite data. These findings underscore the need to interpret remote sensing data cautiously, recognizing both its strengths for long-term monitoring and its constraints in detecting small-scale environmental changes. However, these observed relationships should not be interpreted as direct causal effects and should therefore be interpreted with appropriate caution, given the observational nature of the dataset and the absence of process-based validation through *in-situ* measurements or mechanistic modeling.

In conclusion, this study demonstrates the scientific value of long-term, satellite-based monitoring for aquaculture management, even when no statistically significant impacts are detected. Null findings are essential for defining the limits of remote sensing applications and improving the design of future monitoring frameworks. Integrating high-resolution satellite imagery and *in-situ* observations will be crucial for advancing our understanding of aquaculture–environment interactions and for guiding sustainable marine spatial planning in Türkiye and other countries with established aquaculture industries.

## Supplemental Information

10.7717/peerj.20873/supp-1 Supplemental Information 1 Raw Data for Aquaculture Site from GEE.

10.7717/peerj.20873/supp-2 Supplemental Information 2 Raw Data for Control Site 1 from GEE.

10.7717/peerj.20873/supp-3 Supplemental Information 3 Raw Data for Control Site 2 from GEE.

10.7717/peerj.20873/supp-4 Supplemental Information 4 Robustness check: Difference-in-Differences results using offshore control site (Control 2).

10.7717/peerj.20873/supp-5 Supplemental Information 5 Data extraction code for aquaculture site.

10.7717/peerj.20873/supp-6 Supplemental Information 6 Data extraction code for control site 1.

10.7717/peerj.20873/supp-7 Supplemental Information 7 Data extraction code for control site 2.

10.7717/peerj.20873/supp-8 Supplemental Information 8 Detailed methodology for data processing and analysis.

## Additional Information and Declarations

### Data Availability

The following information was supplied regarding data availability:

The code is available at Zenodo: Tosun, D. D. (2025). Google Earth Engine Code for Seasonal Oceanographic Time Series Extraction (Chlorophyll-a, SST, PAR) Across Three Aquaculture Areas (2002–2022). Zenodo. https://doi.org/10.5281/zenodo.17257007.
