# Supplementary material for: Monitoring environmental impacts of a designated aquaculture area in the Karaburun Peninsula using Google Earth Engine
Source: PeerJ. 2026 Feb 23;14:e20873. doi: 10.7717/peerj.20873 (PMC12939785; doi:10.7717/peerj.20873)
Supplement: Supplemental Information 4 [file peerj-14-20873-s004.docx]

**Title:**
*Robustness check: Difference-in-Differences results using offshore control site (Control 2).*

| **Parameter** | **DiD coefficient** | **Std. Error** | **p-value** | **N** |
| --- | --- | --- | --- | --- |
| Chl-a | -0.0176 | 0.022 | 0.430 | 237 |
| POC | -1.489 | 4.860 | 0.759 | 158 |
| PAR | 0.185 | 13.270 | 0.989 | 158 |

**Footnote (önemli):**

*Heteroscedasticity-robust (HC3) standard errors are reported. The interaction term (treated × post) represents the Difference-in-Differences estimator.*
