# Supplementary material for: Monitoring environmental impacts of a designated aquaculture area in the Karaburun Peninsula using Google Earth Engine
Source: PeerJ. 2026 Feb 23;14:e20873. doi: 10.7717/peerj.20873 (PMC12939785; doi:10.7717/peerj.20873)
Supplement: Supplemental Information 7 [file peerj-14-20873-s007.docx]

/**

* @license

* Copyright 2024 Google LLC.

* SPDX-License-Identifier: Apache-2.0

*/

// Updated with new coordinates for the aquaculture field

var aquacultureField = ee.Geometry.Polygon(

[[[26.433763181138872, 38.68836249114685],

[26.433763181138872, 38.674895072578785],

[26.49599043028438, 38.674895072578785],

[26.49599043028438, 38.68836249114685]]], null, false);

// Define the start and end dates for your time series analysis.

var startDate = '2002-07-04'; // MODIS/Aqua starts July 4, 2002

var endDate = '2022-02-28'; // Adjusted to reflect actual data availability

// --- DATA SELECTION AND FILTERING ---

// 1. MODIS/Aqua Ocean Color SMI: Standard Mapped Image (L3SMI)

var modisOceanProducts = ee.ImageCollection('NASA/OCEANDATA/MODIS-Aqua/L3SMI')

.filterDate(startDate, endDate)

.filterBounds(aquacultureField)

.select(['chlor_a', 'sst', 'poc', 'nflh']);

// 2. MODIS PAR Data (MCD18C2 - Daily 3-Hour PAR)

var pARData = ee.ImageCollection('MODIS/061/MCD18C2')

.filterDate(startDate, endDate)

.filterBounds(aquacultureField);

// Function to calculate daily mean PAR from the 3-hourly data

var calculateDailyPAR = function(image) {

// Select all PAR bands

var parBands = image.select(['GMT_0000_PAR', 'GMT_0300_PAR', 'GMT_0600_PAR',

'GMT_0900_PAR', 'GMT_1200_PAR', 'GMT_1500_PAR',

'GMT_1800_PAR', 'GMT_2100_PAR']);

// Calculate mean across all time steps

var dailyMean = parBands.reduce(ee.Reducer.mean()).rename('PAR_Mean');

// Copy properties from original image

return image.addBands(dailyMean).copyProperties(image, ['system:time_start']);

};

// Calculate daily mean PAR for each image

var pARDataDaily = pARData.map(calculateDailyPAR).select(['PAR_Mean']);

// --- PRELIMINARY INSPECTION ---

print('Number of MODIS Ocean Product images (daily):', modisOceanProducts.size());

print('Number of PAR images (daily):', pARDataDaily.size());

// Add aquaculture field to the map for visual confirmation.

Map.centerObject(aquacultureField, 10);

Map.addLayer(aquacultureField, {color: 'blue'}, 'Aquaculture Field');

// --- SEASONAL AGGREGATION (IMPROVED) ---

// Get a list of all years in the collection.

var years = ee.List.sequence(2002, 2022);

// Define seasons with their corresponding months

var seasons = [

{name: 'Winter', months: [12, 1, 2]},

{name: 'Spring', months: [3, 4, 5]},

{name: 'Summer', months: [6, 7, 8]},

{name: 'Autumn', months: [9, 10, 11]}

];

// Create a function that generates all year-season combinations for Ocean Products

var createSeasonalComposites = function() {

var yearSeasonPairs = [];

// Create all year-season combinations

for (var year = 2002; year <= 2022; year++) {

for (var i = 0; i < seasons.length; i++) {

yearSeasonPairs.push({

year: year,

season: seasons[i]

});

}

}

// Convert to server-side processing

var allImages = yearSeasonPairs.map(function(pair) {

var year = pair.year;

var season = pair.season;

var seasonName = season.name;

var months = season.months;

var yearCollection = modisOceanProducts.filter(ee.Filter.calendarRange(year, year, 'year'));

// Handle winter months that span across years

var seasonCollection;

if (seasonName === 'Winter') {

// For winter, we need December of previous year and Jan-Feb of current year

var prevDecember = modisOceanProducts

.filter(ee.Filter.calendarRange(year - 1, year - 1, 'year'))

.filter(ee.Filter.calendarRange(12, 12, 'month'));

var currentJanFeb = yearCollection

.filter(ee.Filter.calendarRange(1, 2, 'month'));

seasonCollection = prevDecember.merge(currentJanFeb);

} else {

seasonCollection = yearCollection

.filter(ee.Filter.calendarRange(months[0], months[months.length-1], 'month'));

}

// Calculate mean if we have data, otherwise return null image

var composite = ee.Algorithms.If(

seasonCollection.size().gt(0),

seasonCollection.mean().set({

'year': year,

'season': seasonName,

'system:time_start': ee.Date.fromYMD(year, months[0], 1).millis(),

'image_count': seasonCollection.size()

}),

ee.Image([

ee.Image.constant(-9999).rename('chlor_a'),

ee.Image.constant(-9999).rename('sst'),

ee.Image.constant(-9999).rename('poc'),

ee.Image.constant(-9999).rename('nflh')

]).set({

'year': year,

'season': seasonName,

'system:time_start': ee.Date.fromYMD(year, months[0], 1).millis(),

'image_count': 0

})

);

return ee.Image(composite);

});

return ee.ImageCollection(allImages);

};

// Create a function for PAR seasonal composites

var createPARSeasonalComposites = function() {

var yearSeasonPairs = [];

// Create all year-season combinations

for (var year = 2002; year <= 2022; year++) {

for (var i = 0; i < seasons.length; i++) {

yearSeasonPairs.push({

year: year,

season: seasons[i]

});

}

}

// Convert to server-side processing

var allPARImages = yearSeasonPairs.map(function(pair) {

var year = pair.year;

var season = pair.season;

var seasonName = season.name;

var months = season.months;

var yearCollection = pARDataDaily.filter(ee.Filter.calendarRange(year, year, 'year'));

// Handle winter months that span across years

var seasonCollection;

if (seasonName === 'Winter') {

var prevDecember = pARDataDaily

.filter(ee.Filter.calendarRange(year - 1, year - 1, 'year'))

.filter(ee.Filter.calendarRange(12, 12, 'month'));

var currentJanFeb = yearCollection

.filter(ee.Filter.calendarRange(1, 2, 'month'));

seasonCollection = prevDecember.merge(currentJanFeb);

} else {

seasonCollection = yearCollection

.filter(ee.Filter.calendarRange(months[0], months[months.length-1], 'month'));

}

// Calculate mean if we have data, otherwise return null image

var composite = ee.Algorithms.If(

seasonCollection.size().gt(0),

seasonCollection.mean().set({

'year': year,

'season': seasonName,

'system:time_start': ee.Date.fromYMD(year, months[0], 1).millis(),

'par_image_count': seasonCollection.size()

}),

ee.Image([

ee.Image.constant(-9999).rename('PAR_Mean')

]).set({

'year': year,

'season': seasonName,

'system:time_start': ee.Date.fromYMD(year, months[0], 1).millis(),

'par_image_count': 0

})

);

return ee.Image(composite);

});

return ee.ImageCollection(allPARImages);

};

var allSeasonalComposites = createSeasonalComposites();

var allPARSeasonalComposites = createPARSeasonalComposites();

print('Total seasonal composites created:', allSeasonalComposites.size());

print('Total PAR seasonal composites created:', allPARSeasonalComposites.size());

// --- TIME SERIES EXTRACTION FOR AQUACULTURE AREA ---

// Function to extract mean values for each seasonal composite (Ocean products)

var extractSeasonalTimeSeriesForAquaculture = function(image) {

var currentImage = ee.Image(image);

// Reduce the image to get the mean value for each band within the specified geometry

var reduced = currentImage.reduceRegion({

reducer: ee.Reducer.mean(),

geometry: aquacultureField,

scale: 1000, // MODIS data resolution is ~1km

bestEffort: true,

maxPixels: 1e9

});

// Get values from the reduced dictionary

var chlorA_value = reduced.get('chlor_a');

var sst_value = reduced.get('sst');

var poc_value = reduced.get('poc');

var nflh_value = reduced.get('nflh');

// Create a date string for easier sorting/analysis

var year = image.get('year');

var season = image.get('season');

var imageCount = image.get('image_count');

// Return a new Feature with the extracted values

return ee.Feature(null, {

'year': year,

'season': season,

'date_string': ee.String(year).cat('_').cat(season),

'area_type': 'Aquaculture',

'chlor_a_mg_per_m3': chlorA_value,

'sst_celsius': sst_value,

'poc_mg_per_m3': poc_value,

'nflh_mw_per_cm2_per_um_per_sr': nflh_value,

'image_count': imageCount

});

};

// Function to extract PAR values for each seasonal composite

var extractPARTimeSeriesForAquaculture = function(image) {

var currentImage = ee.Image(image);

// Reduce the image to get the mean value for PAR within the specified geometry

var reduced = currentImage.reduceRegion({

reducer: ee.Reducer.mean(),

geometry: aquacultureField,

scale: 5600, // PAR data resolution is ~5.6km

bestEffort: true,

maxPixels: 1e9

});

// Get PAR value from the reduced dictionary

var par_value = reduced.get('PAR_Mean');

// Create a date string for easier sorting/analysis

var year = image.get('year');

var season = image.get('season');

var parImageCount = image.get('par_image_count');

// Return a new Feature with the extracted values

return ee.Feature(null, {

'year': year,

'season': season,

'date_string': ee.String(year).cat('_').cat(season),

'area_type': 'Aquaculture',

'par_mol_per_m2_per_day': par_value,

'par_image_count': parImageCount

});

};

// Apply the extraction functions to the seasonal composite collections

var aquacultureSeasonalTimeSeries = allSeasonalComposites.map(extractSeasonalTimeSeriesForAquaculture);

var parSeasonalTimeSeries = allPARSeasonalComposites.map(extractPARTimeSeriesForAquaculture);

// --- COMBINE OCEAN AND PAR DATA ---

// Create a function to join the ocean and PAR data

var joinOceanAndPAR = function(oceanFeature) {

var oceanFeatureTyped = ee.Feature(oceanFeature);

var year = oceanFeatureTyped.get('year');

var season = oceanFeatureTyped.get('season');

// Find matching PAR feature

var matchingPAR = parSeasonalTimeSeries.filter(

ee.Filter.and(

ee.Filter.equals('year', year),

ee.Filter.equals('season', season)

)

).first();

var parValue = ee.Algorithms.If(

ee.Algorithms.IsEqual(matchingPAR, null),

-9999,

matchingPAR.get('par_mol_per_m2_per_day')

);

var parImageCount = ee.Algorithms.If(

ee.Algorithms.IsEqual(matchingPAR, null),

0,

matchingPAR.get('par_image_count')

);

// Return combined feature

return oceanFeatureTyped.set({

'par_mol_per_m2_per_day': parValue,

'par_image_count': parImageCount

});

};

// Apply the join function

var combinedTimeSeries = aquacultureSeasonalTimeSeries.map(joinOceanAndPAR);

// --- VISUALIZATION AND EXPORT ---

print('Combined Seasonal Time Series Data size:', combinedTimeSeries.size());

// Print a sample of the extracted features for debugging

print('Sample Combined Seasonal Time Series Features (first 5):',

combinedTimeSeries.limit(5));

// Sort the collection by year and season for better organization

var sortedCombinedTimeSeries = combinedTimeSeries.sort('date_string');

// Export the combined aquaculture field time series data to a CSV file

Export.table.toDrive({

collection: sortedCombinedTimeSeries,

description: 'Aquaculture_Seasonal_TimeSeries_Data_With_PAR',

folder: 'GEE_Mariculture_Analysis',

fileNamePrefix: 'aquaculture_seasonal_data_with_par',

fileFormat: 'CSV'

});

// Additional export with filtered data (only records with actual data)

var filteredCombinedTimeSeries = sortedCombinedTimeSeries.filter(

ee.Filter.and(

ee.Filter.neq('image_count', 0),

ee.Filter.neq('par_image_count', 0)

)

);

Export.table.toDrive({

collection: filteredCombinedTimeSeries,

description: 'Aquaculture_Seasonal_TimeSeries_Data_With_PAR_Filtered',

folder: 'GEE_Mariculture_Analysis',

fileNamePrefix: 'aquaculture_seasonal_data_with_par_filtered',

fileFormat: 'CSV'

});

// --- VISUALIZATION: Add sample composites to the map ---

var sampleComposite = ee.Image(allSeasonalComposites.first());

var samplePARComposite = ee.Image(allPARSeasonalComposites.first());

Map.addLayer(sampleComposite.select('chlor_a'),

{min: 0, max: 5, palette: ['blue', 'cyan', 'yellow', 'red']},

'Sample Chlorophyll-a');

Map.addLayer(samplePARComposite.select('PAR_Mean'),

{min: 0, max: 60, palette: ['purple', 'blue', 'cyan', 'yellow', 'red']},

'Sample PAR');

print('Script completed successfully!');

print('Remember to run the export tasks from the Tasks tab to download your data.');

print('PAR data units: mol photons/m²/day');

print('PAR data resolution: ~5.6km vs Ocean data resolution: ~1km');
