# Supplementary material for: Monitoring environmental impacts of a designated aquaculture area in the Karaburun Peninsula using Google Earth Engine
Source: PeerJ. 2026 Feb 23;14:e20873. doi: 10.7717/peerj.20873 (PMC12939785; doi:10.7717/peerj.20873)
Supplement: Supplemental Information 8 [file peerj-14-20873-s008.docx]

**Supplementary Material — Data Processing and Analysis Workflow**

**S1.1 Software Environment**

All data processing and statistical analyses were performed using:

- **Python 3.13.4**
- **Jupyter Lab 4.4.4**

The following libraries were used:

- **Pandas 2.3.0** – data manipulation
- **NumPy 2.3.0** – numerical operations
- **Matplotlib 3.10.3** and **Seaborn 0.13.2** – data visualization
- **SciPy 1.16.0** – regression and statistics
- **Statsmodels 0.14.5** – ANOVA and Difference-in-Differences models

**S1.2 Loading and Preparation of GEE Export Files**

CSV files exported from Google Earth Engine for the aquaculture and control sites were:

1. Imported as **Pandas DataFrames**.
2. Purged of initial generic header rows included by GEE.
3. Assigned descriptive variable names consistent across all sites (e.g.,
   'chlor_a_mg_per_m3', 'sst_celsius', 'year', 'season').

**S1.3 Data Cleaning and Type Conversion**

- All water quality parameters and the *year* field were converted to numeric data types.
- Non-numeric entries caused by GEE export artifacts were coerced to **NaN**.
- Rows containing NaN in critical analytical fields were removed to ensure dataset integrity.

**S1.4 Site Classification and Merging**

Each dataset was tagged with an 'area_type' field identifying:

- **Aquaculture**,
- **Control_1 (coastal)**,
- **Control_2 (offshore)**

The DataFrames were then concatenated into a unified dataset for analysis.

**S1.5 Temporal Variable Construction**

To support seasonal and long-term analyses:

- A combined 'time_period' variable was created by merging *year* and *season* into a consistent label (e.g., "2015-Winter").
- This was converted into a datetime-like object for chronological sorting and plotting.
